# NBA stars’ China tours and college students’ sports consumption intention: the mediating role of perceived value

**DOI:** 10.3389/fspor.2026.1920395

**Published:** 2026-08-31

**Authors:** Yunfei Wang, Haojie Zhang, Jiayu Li, Xiaoping Jiang

**Affiliations:** 1 College of Physical Education and Health Sciences, Zhejiang Normal University, Jinhua, China; 2 College of Teacher Education, Zhejiang Normal University, Jinhua, China

**Keywords:** college students, elaboration likelihood model, NBA stars' China tours, perceived value, S-O-R theory, sports consumption intention

## Abstract

This study examined the associations between selected cues encountered in the context of NBA stars’ China tours and college students’ sports consumption intention, and tested the hypothesized mediating role of perceived value. Drawing on Stimulus-Organism-Response (S-O-R) theory and the Elaboration Likelihood Model (ELM), this study constructed a mediation model in which information quality, players’ professional achievements, and players’ personal charisma were treated as antecedent variables, perceived value as the mediating variable, and sports consumption intention as the outcome variable. A cross-sectional questionnaire survey was conducted among 1,024 college students in Chongqing. Reliability and validity tests and Pearson correlation analysis were performed, followed by an integrated path analysis in AMOS 28.0 in which the three antecedent variables were entered simultaneously. Specific indirect associations through perceived value were evaluated using 5,000 bias-corrected bootstrap resamples. The results showed significant positive correlations among information quality, players’ professional achievements, players’ personal charisma, perceived value, and sports consumption intention. In the integrated model, information quality, players’ professional achievements, and players’ personal charisma were each positively associated with perceived value and sports consumption intention, while perceived value was positively associated with sports consumption intention. Significant indirect associations through perceived value were observed for information quality [*B* = 0.090, 95% BC CI (0.068, 0.115)], players’ professional achievements [*B* = 0.126, 95% BC CI (0.089, 0.169)], and players’ personal charisma [*B* = 0.183, 95% BC CI (0.128, 0.246)]. Demographic variables showed no significant differences in sports consumption intention. These findings suggest that perceived value mediates the associations between perceived tour-related attributes and sports consumption intention among the surveyed college students in Chongqing.

## 1 Introduction

The National Basketball Association (NBA), one of the most influential professional sports leagues worldwide, has gradually developed a sports-cultural communication and commercial marketing system tailored to the Chinese market through game broadcasting, athlete promotion, commercial partnerships, cultural adaptation, and localized marketing (1). In this study, “NBA stars’ China tours” refer to basketball promotion, fan interaction, brand communication, public-welfare engagement, and cultural-exchange activities conducted for Chinese audiences by current or retired NBA players under the organization or sponsorship of the NBA, sports brands, commercial institutions, or other relevant organizations. Rather than constituting a single form of commercial promotion, these tours represent comprehensive sport communication events. Unlike conventional celebrity endorsement, which primarily transfers the cultural meanings associated with celebrities to products (2), NBA stars’ China tours combine in-person appearances, real-time interaction, and commercial communication, thereby providing consumers with a specific context in which to evaluate the participating athletes as well as the information, activities, products, and experiences surrounding the tours.

Existing sport consumption research on the context of NBA stars’ China tours remains limited. Nevertheless, related studies provide theoretical support for examining the associations between tour-related cues and college students’ sports consumption intention. Previous research has indicated that positive attitudes toward participation in sports activities are consistently associated with college students’ sport consumption intention across different consumption types and communication channels (3). Athlete-brand research further suggests that consumers evaluate athletes on the basis of both on-field and off-field attributes. More specifically, athlete brand image encompasses athletic performance, attractive appearance, and a marketable lifestyle, indicating that both professional achievements and personal characteristics constitute important bases for consumers’ evaluations of athletes (4). Research on sporting events has also shown that large-scale sporting events may enhance participants’ psychological benefits and, in turn, strengthen their intentions to attend events and purchase licensed merchandise (5). Drawing on these related strands of research, the present study conceptualizes NBA stars’ China tours as an overarching external sport-marketing stimulus and research context rather than as a binary indicator of on-site participation. Consumers may encounter this stimulus through both in-person and mediated forms of contact, such as tour-related information, livestreams, social media content, and other forms of communication. Within this broader context, consumers’ perceptions of the tour stimulus are operationalized through three focal cues: information quality, players’ professional achievements, and players’ personal charisma. These cues reflect the communication-related and athlete-related attributes that consumers encounter in connection with the tours and may be associated with their subsequent sports consumption responses.

Sports consumption intention reflects an individual's psychological readiness and likelihood of engaging in sport-related consumption behaviors (6). Because the present study does not observe actual consumption behavior, the behavioral response is operationalized as NBA-related sports consumption intention. Specifically, this outcome is measured through three dimensions—participation intention, recommendation intention, and premium purchase intention—which respectively reflect college students’ willingness to participate in NBA-related activities, recommend related activities or products to others, and purchase related products or services at a premium (7–9). Therefore, sports consumption intention serves as the outcome variable through which the present study assesses college students’ behavioral responses to cues related to NBA stars’ China tours.

However, exposure to information about NBA stars’ China tours or favorable evaluations of participating players may not necessarily translate directly into intentions to participate, purchase, recommend, or pay a price premium. Consumers’ judgments regarding whether participation in related activities, the purchase of related products, or the acquisition of related experiences is worthwhile constitute an important basis for the formation of behavioral intentions. Previous research in sport consumption contexts has shown that perceived value is closely associated with consumers’ intentions to participate in activities and purchase sport-related products and may serve as an important psychological mechanism linking consumers’ prior evaluations with subsequent behavioral intentions (10–12). Therefore, examining the relationship between NBA stars’ China tour-related cues and sport consumption intention also requires attention to how consumers translate these external cues into overall value evaluations of the associated activities, products, and experiences.

Although direct empirical research on the role of perceived value in the specific context of NBA stars’ China tours remains very limited, related sport management research provides an important basis for introducing perceived value into this research context. Previous research has shown that the development of the NBA in the Chinese market has long relied on localized promotion, athlete-related communication, and interactive marketing activities targeting fans (1). From the broader perspective of professional sport consumption, consumers do not form behavioral intentions solely on the basis of the core sport product itself, but also evaluate the interaction process, activity environment, and overall sport experience. Clemes et al. showed that professional sport spectators’ evaluations of interaction quality, physical environment quality, and outcome quality were associated with their perceived value and satisfaction and, in turn, with subsequent behavioral intentions (13). In a professional basketball consumption context, Calabuig Moreno et al. further found that service quality significantly predicted spectators’ perceived value, which in turn was an important predictor of future behavioral intentions (14). Together with findings from perceived-value research in sport consumption, these studies suggest that consumers translate different external cues and experiences in sport-related activities into overall value judgments, which are subsequently associated with behavioral intentions (11, 12). Therefore, incorporating perceived value into research on NBA stars’ China tours has a clear foundation in the sport consumption literature.

**Figure 1:**
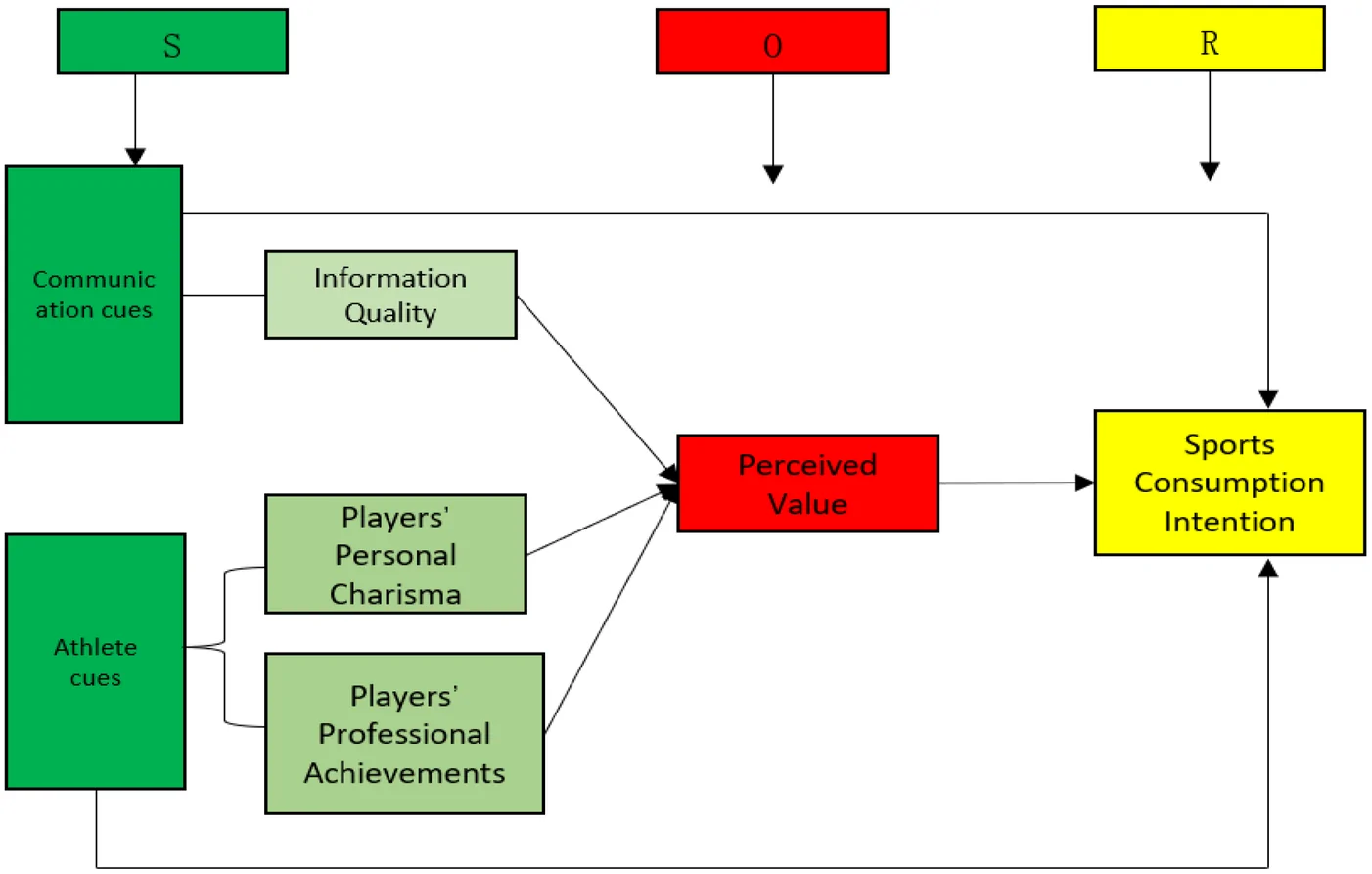
Research hypothesis model.

To address the above research gaps, the present study further examines whether more favorable evaluations of tour-related cues among college students are associated with stronger sports consumption intention, and whether perceived value plays the hypothesized mediating role in these relationships. The Stimulus-Organism-Response (S-O-R) framework is adopted as the primary theoretical structure for specifying the relationships among external tour-related cues, perceived value, and sports consumption intention, while the Elaboration Likelihood Model (ELM) provides a complementary theoretical basis for distinguishing communication-related message cues from athlete-related source cues (15, 16). On this basis, the study develops an analytical framework of “external tour-related stimuli → perceived value → sports consumption intention (Figure 1).”

### 1.1 Theoretical framework

The S-O-R framework provides the primary theoretical basis for organizing the relationships examined in this study. According to the framework, environmental stimuli are associated with individuals’ internal cognitive and affective states, which are subsequently associated with behavioral responses (15). Applied to the context of NBA stars’ China tours, information quality, players’ professional achievements, and players’ personal charisma are conceptualized as three external stimuli (S) encountered or evaluated by consumers. Perceived value represents the organism (O) because it captures consumers’ internal evaluation of the benefits obtained from tour-related activities, products, and experiences relative to the costs involved. Sports consumption intention represents the response (R), reflecting consumers’ willingness to participate, recommend, and make premium purchases in relation to NBA-related activities and offerings. Thus, the basic theoretical structure of the present study is expressed as external tour-related stimuli (S) → perceived value (O) → sports consumption intention (R).

The positioning of perceived value as the organism is consistent with the role assigned to internal evaluative states in the S-O-R framework. Perceived value reflects consumers’ cognitive evaluation of whether the functional, emotional, and social benefits obtained from an activity, product, or experience are worthwhile relative to the monetary and non-monetary costs involved. Previous S-O-R research has similarly treated customer perceived value as an internal psychological mechanism linking environmental stimuli with subsequent behavioral intention (17). Accordingly, perceived value is expected to represent an internal evaluative state through which consumers interpret tour-related external cues before forming sports consumption intentions. Given that the present study focuses on consumers’ overall evaluations of tour-related activities, products, and experiences rather than on a single standardized product with a uniform price, perceived value is operationalized in terms of functional value, emotional value, and social value, while price value is not treated as a separate dimension.

ELM complements the S-O-R framework by providing a basis for distinguishing qualitatively different external cues. ELM suggests that persuasive judgments may draw on information contained in a message as well as cues associated with the information source (16). In the present study, information quality represents a communication-related message cue because it concerns the completeness, timeliness, customization, and scope of information surrounding NBA stars’ China tours. In contrast, players’ professional achievements and personal charisma represent athlete-related source cues because they concern characteristics of the participating athletes themselves. ELM is therefore used to explain the inclusion and classification of different types of external stimuli, whereas the S-O-R framework specifies how these stimuli are theoretically associated with perceived value and sports consumption intention. Because the present study does not measure consumers’ level of elaboration or directly test central- and peripheral-route processing, ELM is not treated as a direct test of dual-route information processing.

On the basis of these complementary theoretical roles, the proposed hypotheses follow the S-O-R structure. The associations between the three external stimuli and perceived value represent the stimulus-to-organism relationships (H1a–H1c). The association between perceived value and sports consumption intention represents the organism-to-response relationship (H2). In addition, prior sport-consumer and athlete-brand research provides a basis for examining whether the three external cues are also directly associated with sports consumption intention (H3a–H3c). Most importantly, the S-O-R sequence provides the theoretical basis for examining perceived value as an intervening evaluative mechanism linking each external stimulus with sports consumption intention (H4a–H4c). ELM complements these relationships by distinguishing information quality as a communication-related message cue from professional achievements and personal charisma as athlete-related source cues. The specific direction of each hypothesized association is further developed below on the basis of construct-specific theory and empirical evidence.

### 1.2 Hypothesis development

Information quality provides consumers with an important basis for evaluating the potential benefits of an activity or service. The DeLone and McLean Information Systems Success Model identifies information quality as an important factor shaping users’ subsequent evaluations (18). In a sport-branded app context, Won et al. found that higher information quality was associated with stronger perceptions of usefulness, ease of use, and enjoyment (19). In the context of NBA stars’ China tours, higher-quality information in terms of completeness, timeliness, customization, and scope may reduce uncertainty regarding tour arrangements, participation procedures, and potential benefits, thereby facilitating more favorable value evaluations. Therefore, higher information quality is expected to be associated with higher perceived value. Accordingly:

H1a: Information quality is positively associated with perceived value.

Players’ professional achievements provide consumers with important information regarding athletes’ competence and sport-related status. Athletic performance has been identified as a core dimension of athlete brand image (4), while research on star-player characteristics has also highlighted the relevance of athletes’ on-field attributes to consumer evaluations of professional sport brands (20). In the context of NBA stars’ China tours, stronger professional achievements may signal greater expertise, competence, and prestige, thereby contributing to more favorable overall value evaluations of the participating athletes and related activities. Therefore, as consumers evaluate participating players as more professionally accomplished, their perceived value of the related activities and offerings is expected to increase. Accordingly:

H1b: Players’ professional achievements are positively associated with perceived value.

Unlike professional achievements, personal charisma reflects consumers’ evaluations of athletes’ personality, attractiveness, affinity, and interpersonal appeal. Athlete-brand research indicates that consumers evaluate athletes not only on the basis of athletic performance but also through personal and off-field attributes (4). Research on sport celebrities further suggests that celebrity-related appeal and experiential factors are relevant to consumers’ expected value evaluations (21). In the context of NBA stars’ China tours, stronger personal charisma may therefore enhance anticipated enjoyment, psychological closeness, and emotional and social benefits. Therefore, stronger perceived personal charisma is expected to be associated with higher perceived value. Accordingly:

H1c: Players’ personal charisma is positively associated with perceived value.

Perceived value has been consistently associated with subsequent behavioral intentions across different sport consumption contexts. Wang et al. found that perceived value mediated the relationships between price and quality perceptions and marathon participation intention (10). Kwon et al. demonstrated its mediating role between team identification and purchase intention for team-licensed apparel (12), whereas Shapiro et al. reported a close relationship between perceived value and purchase intention in pay-per-view sport consumption (11). Research on professional team sport and professional basketball has likewise demonstrated the importance of perceived value for subsequent consumption behavior and future intentions (14, 22). In the present context, when consumers perceive that NBA tour-related activities, products, and experiences provide greater functional, emotional, and social benefits relative to the monetary and non-monetary costs involved, they are more likely to regard such consumption as worthwhile. A stronger sense that the related activities and offerings are worthwhile should, in turn, be accompanied by greater willingness to participate, recommend, and make premium purchases. Therefore, higher perceived value is expected to be associated with stronger sports consumption intention. Accordingly:

H2: Perceived value is positively associated with NBA-related sports consumption intention.

Information quality may also be positively associated with sports consumption intention independently of its association through perceived value. Complete, timely, relevant, and clear tour information can reduce uncertainty regarding event schedules, participation procedures, access requirements, and related offerings, thereby making consumption opportunities easier to understand and evaluate. Sport-related digital research has similarly linked higher information quality with more favorable usage-related evaluations (19). When consumers have clearer and more reliable information about how and why to participate, they may feel more confident in making participation and consumption decisions. Therefore, higher information quality is expected to be associated with stronger sports consumption intention. Accordingly:

H3a: Information quality is positively associated with NBA-related sports consumption intention.

Players’ professional achievements may also be positively associated with sports consumption intention. Athletic performance and professional competence constitute important components of athlete brand evaluations (4, 20). In the context of NBA stars’ China tours, stronger professional achievements may signal greater competence, credibility, and sport-related prestige, thereby increasing the attractiveness of the participating athletes and the activities or offerings associated with them. Consumers who evaluate participating players as more professionally accomplished may consequently be more willing to participate in related activities, recommend them to others, or purchase related offerings. Therefore, higher evaluations of players’ professional achievements are expected to be associated with stronger sports consumption intention. Accordingly:

H3b: Players’ professional achievements are positively associated with NBA-related sports consumption intention.

Players’ personal charisma may likewise be positively associated with sports consumption intention. Personal and off-field athlete attributes can contribute to favorable evaluations of athlete brands (4), while sport-celebrity research suggests that personal appeal and related experiential responses are relevant to consumer evaluations (21). In the context of NBA stars’ China tours, stronger perceived charisma may increase consumers’ affinity toward the participating player, anticipated enjoyment of athlete-related interactions, and willingness to engage with activities and offerings associated with that player. Consequently, consumers who perceive stronger personal charisma may show greater willingness to participate, recommend, or make premium purchases. Therefore, players’ personal charisma is expected to be positively associated with sports consumption intention. Accordingly:

H3c: Players’ personal charisma is positively associated with NBA-related sports consumption intention.

The hypothesized mediating role of perceived value is grounded primarily in the S-O-R framework, according to which external stimuli are associated with behavioral responses through consumers’ internal cognitive and affective states (15). Sport consumer research provides empirical support for this general mechanism. Byon et al. found that perceived value mediated the relationships between core and peripheral service quality and professional team sport consumption behavior (22). Kwon et al. similarly demonstrated the mediating role of perceived value between team identification and purchase intention for team-licensed apparel (12), while Shapiro et al. found that perceived value partially mediated the relationship between identification and purchase intention in pay-per-view sport consumption (11). In the present study, the expected indirect associations are positive because each external cue is theorized to contribute to a more favorable value evaluation, and higher perceived value is, in turn, expected to be associated with stronger sports consumption intention. Specifically, higher information quality may reduce uncertainty and thereby increase perceived value; stronger professional achievements may enhance favorable evaluations of the participating athletes and related activities; and stronger personal charisma may increase anticipated enjoyment, psychological closeness, and other valued benefits. Because these more favorable value evaluations are expected to accompany stronger participation, recommendation, and premium-purchase intentions, perceived value is expected to transmit a positive indirect association from each external cue to sports consumption intention. Accordingly:

H4a: Perceived value mediates the association between information quality and NBA-related sports consumption intention.
H4b: Perceived value mediates the association between players’ professional achievements and NBA-related sports consumption intention.
H4c: Perceived value mediates the association between players’ personal charisma and NBA-related sports consumption intention.

Because previous studies have reported inconsistent findings regarding demographic differences in sports consumption and no clear directional expectations can be established, the relevant variables are examined exploratorily:

RQ1: Does NBA-related sports consumption intention differ across demographic, socioeconomic, and basketball-related characteristics among college students?

## 2 Materials and methods

### 2.1 Participants

This study focused on college students in Chongqing and selected six representative universities in Chongqing, including Chongqing Normal University, Yangtze Normal University, Chongqing Technology and Business University, Southwest University, Chongqing University, and Southwest University of Political Science and Law, to distribute questionnaires. From June 8th to 14th, 2026, questionnaires were collected through a combination of offline paper-based distribution and online distribution assisted by Wenjuanxing. A total of 1200 questionnaires were distributed, and 1,121 questionnaires were collected, with a recovery rate of 93.42%. After excluding invalid questionnaires with incomplete responses, random answers, or patterned responses, a total of 1,024 valid questionnaires were obtained, with an effective rate of 91.35%.

### 2.2 Procedure

This study adopted a cross-sectional design and used a multistage purposive and convenience sampling procedure to recruit students from six universities in Chongqing. Although a cross-sectional design cannot establish temporal precedence or causal relationships, it was appropriate at the exploratory stage of the present study because it enabled the simultaneous examination of theoretically specified associations and indirect effects among multiple constructs in a relatively large sample. The selected universities represented different institutional types, including normal, comprehensive, business, and political and law universities. All students read the informed consent form before participation and were informed that participation was completely voluntary and that they had the right to withdraw at any time. Only respondents who reported prior awareness of NBA stars’ tours in China were eligible to complete the main questionnaire. Before entering the main questionnaire, respondents completed a screening item assessing whether they were aware of NBA stars’ tours in China. Only respondents who selected “YES” were allowed to proceed to the main questionnaire. The awareness screening item was used to ensure that respondents had a minimum level of familiarity with the research context rather than to quantify the intensity of their exposure or to require prior on-site participation. Awareness of NBA stars’ China tours could arise through either direct participation or mediated exposure to tour-related content. Accordingly, on-site attendance was not an eligibility requirement in the present study.

After the survey, the researchers collected, organized, and screened the questionnaires. The criteria for invalid questionnaires included more than 20% missing responses, obvious repetitive answering patterns, or inconsistent answers. After the final review, the researchers entered the valid data into the system. All the above procedures complied with the ethical standards of scientific research and were approved in advance by the Ethics Review Committee of Zhejiang Normal University, with the approval number ZSRT2026201.

### 2.3 Variables and measurement instruments

To measure the five focal constructs, the questionnaire drew on established scales and related studies cited below and adapted their content to the context of NBA stars’ China tours. The adaptation primarily involved contextualizing the item wording and selecting dimensions relevant to tour-related information, participating players, activities, products, and experiences, with the aim of retaining the conceptual content of the source constructs. The final questionnaire included five core variables: information quality, players’ professional achievements, players’ personal charisma, perceived value, and sports consumption intention. All items were measured on a five-point Likert scale ranging from 1 (“strongly disagree”) to 5 (“strongly agree”). The complete final item wording and corresponding scale sources are provided in the Supplementary Material.

#### 2.3.1 Selection of measurement instruments

The information quality scale mainly refers to the DeLone and McLean Information Systems Success Model (18), the information quality measurement of online customer satisfaction by McKinney et al. (23), and the integrated study of information quality and technology acceptance by Wixom and Todd (24). Combined with the characteristics of information dissemination in NBA stars’ China tours, four dimensions, namely completeness, timeliness, customization, and scope, were set, with a total of 10 items.

The conceptualization of the two athlete-related variables was informed primarily by research on athlete brand image and star-player characteristics (4, 20), while the China-market context was informed by Zhou et al. (1). The players’ professional achievements scale comprised five items covering two dimensions: achievement cognition and technical ability and professionalism. Players’ personal charisma was measured as a unidimensional construct using three items.

The perceived value scale mainly refers to the consumer perceived value scale proposed by Sweeney and Soutar (25), as well as related studies on the influence of perceived value on behavioral intention in service consumption and sports consumption. Three dimensions, namely functional value, emotional value, and social value, were set, with a total of 10 items. The original perceived value framework was adapted to the context of NBA stars’ China tours. Functional, emotional, and social value were retained because they were theoretically relevant to tour-related activities, products, and experiences.

The sports consumption intention scale mainly refers to the Theory of Planned Behavior, studies on service-quality behavioral outcomes, and research on sports consumption behavioral intention (7–9), and was adapted to the context of NBA stars’ China tours. Consistent with the conceptualization of the behavioral outcome in the Introduction, NBA-related sports consumption intention was operationalized through three dimensions: recommendation intention, participation intention, and premium purchase intention, with a total of nine items.

#### 2.3.2 Reliability and validity tests

Internal consistency was assessed using Cronbach's *α*. The Cronbach's *α* coefficients were 0.856 for information quality, 0.798 for players’ professional achievements, 0.819 for players’ personal charisma, 0.866 for perceived value, and 0.869 for sports consumption intention, all exceeding 0.700. The KMO values ranged from 0.717 to 0.895, and Bartlett's tests of sphericity were statistically significant for all scales.

Confirmatory factor analysis (CFA) was conducted using AMOS 28.0 to assess measurement-model fit and construct validity. The measurement models for information quality, players’ professional achievements, perceived value, and sports consumption intention demonstrated satisfactory fit, with CMIN/DF values ranging from 1.238 to 1.538, RMSEA values from 0.015 to 0.023, RMR values from 0.008 to 0.012, GFI values from 0.992 to 0.998, NFI values from 0.989 to 0.997, RFI values from 0.984 to 0.991, and TLI values from 0.996 to 0.997. Because players’ personal charisma was measured using three items, its single-factor model was just identified; therefore, global model-fit indices were not independently interpreted for this construct.

Convergent validity was assessed using standardized factor loadings, average variance extracted (AVE), and composite reliability (CR). Across the measurement dimensions, standardized factor loadings ranged from 0.691 to 0.823 and AVE values ranged from 0.509 to 0.645. CR values ranged from 0.674 to 0.853; all dimensions exceeded 0.700 except the two-item functional-value dimension (CR = 0.674), for which the AVE was 0.509 and the standardized factor loadings were 0.691 and 0.735. For the multidimensional scales, discriminant validity among the component dimensions was assessed using the Fornell–Larcker criterion, whereby the square root of the AVE for each dimension should exceed its correlations with the other dimensions. The dimensions of information quality, players’ professional achievements, perceived value, and sports consumption intention all satisfied this criterion.

To further assess discriminant validity among the five focal constructs in the research model, the heterotrait–monotrait ratio of correlations (HTMT), as proposed by Henseler et al. (26), was additionally examined. The HTMT values among information quality, players’ professional achievements, players’ personal charisma, perceived value, and sports consumption intention ranged from 0.147 to 0.553. The highest value was 0.553 between perceived value and sports consumption intention, and all values were below the conservative threshold of 0.85, providing further evidence of adequate discriminant validity among the five focal constructs. Detailed results for model fit, convergent validity, and discriminant validity are provided in the Supplementary Material.

### 2.4 Statistical analysis

Data were analyzed using SPSS 27.0 and AMOS 28.0. SPSS 27.0 was used for descriptive statistics, reliability analysis, KMO and Bartlett's tests, common method bias and multicollinearity tests, group-difference analyses, and Pearson correlation analysis. AMOS 28.0 was used for confirmatory factor analysis and for estimating the integrated path model.

The integrated path model was estimated using the composite scores of information quality, players’ professional achievements, players’ personal charisma, perceived value, and NBA-related sports consumption intention. Information quality, players’ professional achievements, and players’ personal charisma were entered simultaneously as exogenous predictors, and their covariances were freely estimated. Perceived value was regressed on all three external cues, while sports consumption intention was regressed on perceived value and the three external cues simultaneously. H1a–H1c were evaluated using the corresponding paths from the three external cues to perceived value, H2 using the path from perceived value to sports consumption intention, and H3a–H3c using the corresponding direct paths from the three external cues to sports consumption intention.

H4a–H4c were evaluated using the specific indirect associations through perceived value. Their statistical significance was assessed using 5,000 bias-corrected bootstrap resamples, with an indirect association considered statistically significant when its 95% confidence interval did not include zero. Standardized coefficients were reported for individual paths, whereas unstandardized coefficients were reported for the specific indirect associations. The explained variance (R^2^) of perceived value and sports consumption intention was also reported. Because the integrated observed-variable path model included all hypothesized direct paths and freely estimated covariances among the three exogenous variables, it was saturated; therefore, global model-fit indices were not interpreted.

## 3 Results

### 3.1 Analysis of survey respondents

The sample was predominantly male (58.79%), and 81.05% of respondents reported an interest in basketball. Stephen Curry was the most frequently preferred player, selected by 34.57% of respondents. In addition, 68.16% of respondents reported a monthly allowance of RMB 1,000–2,000, while 71.48% reported monthly sports expenditure below RMB 300. The full demographic characteristics of the sample are presented in Table 1. The results of the subsequent analyses are presented in Tables 2–8.

**Table 1:** Basic information of the survey respondents.

Variable	Category	Frequency (*N*)	Percentage (%)
Gender	Male	602	58.79
	Female	422	41.21
Grade	Freshman	280	27.34
	Sophomore	336	32.81
	Junior	255	24.90
	Senior	135	13.18
	Postgraduate	18	1.76
Place of Origin	Urban	542	52.93
	Rural	482	47.07
Major	Science and Engineering	488	47.66
	Humanities	298	29.10
	Arts	158	15.43
	Sports	80	7.81
Interest in Basketball	Yes	830	81.05
	No	194	18.95
Favorite Player	Stephen Curry	354	34.57
	LeBron James	274	26.76
	James Harden	134	13.09
	Others	262	25.59
Monthly allowance (RMB)	<1,000	99	9.67
	1,000–1,500	315	30.76
	1,500–2,000	383	37.40
	2,000–2,500	163	15.92
	>2,500	64	6.25
Monthly sports expenditure (RMB)	<100	358	34.96
	100–300	374	36.52
	300–500	235	22.95
	>500	57	5.57

**Table 2:** Results of the common method bias test.

Component	Initial eigenvalues			Extraction sums of squared loadings		
	Total	Variance (%)	Cumulative (%)	Total	Variance (%)	Cumulative (%)
1	8.667	23.424	23.424	8.667	23.424	23.424
2	3.212	8.681	32.105	3.212	8.681	32.105
3	2.416	6.531	38.636	2.416	6.531	38.636
4	2.316	6.260	44.896	2.316	6.260	44.896
5	1.825	4.932	49.828	1.825	4.932	49.828
6	1.323	3.575	53.403	1.323	3.575	53.403
7	1.306	3.530	56.933	1.306	3.530	56.933
8	1.185	3.204	60.137	1.185	3.204	60.137
9	1.152	3.114	63.251	1.152	3.114	63.251
10	1.061	2.869	66.120	1.061	2.869	66.120

**Table 3:** Results of the multicollinearity test.

Variable	Collinearity statistics	
	Tolerance	VIF
IQ	0.872	1.146
PPA	0.860	1.162
PPC	0.894	1.118
PV	0.795	1.257

**Table 4:** Difference analysis.

Variable	Category	SCI (M ± SD)	Test results
Gender	Male	31.21 ± 5.298	t = −0.713 *p* = 0.476
	Female	31.45 ± 5.174	
Grade	Freshman	31.28 ± 5.368	F = 0.719 *p* = 0.579
	Sophomore	31.16 ± 5.356	
	Junior	31.25 ± 5.064	
	Senior	31.96 ± 5.165	
	Postgraduate	30.44 ± 5.575	
Place of origin	Urban	31.26 ± 5.257	t = −0.350 *p* = 0.727
	Rural	31.37 ± 5.239	
Major	Science and Engineering	31.41 ± 5.375	F = 0.478 *p* = 0.697
	Humanities	31.34 ± 5.139	
	Arts	30.86 ± 4.782	
	Sports	31.50 ± 5.748	
Interest in Basketball	Yes	31.36 ± 5.189	t = 0.627 *p* = 0.531
	No	31.10 ± 5.490	
Favorite Player	Stephen Curry	31.17 ± 5.152	F = 1.711 *p* = 0.163
	LeBron James	30.86 ± 5.326	
	James Harden	31.67 ± 5.390	
	Others	31.79 ± 5.292	
Monthly allowance (RMB)	<1,000	30.52 ± 5.292	F = 1.408 *p* = 0.229
	1,000–1,500	31.33 ± 5.108	
	1,500–2,000	31.15 ± 5.294	
	2,000–2,500	31.84 ± 5.081	
	>2,500	32.06 ± 4.998	
Monthly sports expenditure (RMB)	<100	31.26 ± 5.016	F = 0.627 *p* = 0.598
	100–300	31.55 ± 5.215	
	300–500	30.97 ± 5.133	
	>500	31.49 ± 7.084	

All *p*-values are greater than 0.05, indicating no significant differences.

**Table 5:** Correlation analysis.

Variable	IQ	PPA	PPC	PV	SCI
IQ	1				
PPA	0.231**	1			
PPC	0.123**	0.242**	1		
PV	0.330**	0.311**	0.281**	1	
SCI	0.349**	0.298**	0.230**	0.480**	1

**indicates *p* < 0.01.

**Table 6:** Results of the integrated path model.

Hypothesis	Path	B	SE	C.R.	β	*p*
H1a	IQ → PV	0.240	0.027	9.015	0.259	<0.001
H1b	PPA → PV	0.338	0.049	6.880	0.202	<0.001
H1c	PPC → PV	0.491	0.071	6.949	0.200	<0.001
H2	PV → SCI	0.373	0.031	12.066	0.356	<0.001
H3a	IQ → SCI	0.189	0.027	6.880	0.194	<0.001
H3b	PPA → SCI	0.218	0.050	4.388	0.124	<0.001
H3c	PPC → SCI	0.195	0.072	2.720	0.076	0.007

B = unstandardized coefficient; β = standardized coefficient; C.R., critical ratio. R^2^ = 0.205 for perceived value and 0.293 for sports consumption intention.

**Table 7:** Bias-corrected bootstrap tests of specific indirect associations.

Hypothesis	Indirect path	Effect	95% BC LLCI	95% BC ULCI	Decision
H4a	IQ→PV→SCI	0.090	0.068	0.115	Supported
H4b	PPA → PV → SCI	0.126	0.089	0.169	Supported
H4c	PPC → PV → SCI	0.183	0.128	0.246	Supported

Indirect effects are unstandardized estimates. Bias-corrected 95% confidence intervals were based on 5,000 bootstrap resamples.

**Table 8:** Summary of hypothesis testing results.

Hypothesis	Proposed relationship	Statistical result	Decision
H1a	IQ → PV (+)	β = 0.259, *p* < 0.001	Supported
H1b	PPA → PV (+)	β = 0.202, *p* < 0.001	Supported
H1c	PPC → PV (+)	β = 0.200, *p* < 0.001	Supported
H2	PV → SCI (+)	β = 0.356, *p* < 0.001	Supported
H3a	IQ → SCI (+)	β = 0.194, *p* < 0.001	Supported
H3b	PPA → SCI (+)	β = 0.124, *p* < 0.001	Supported
H3c	PPC → SCI (+)	β = 0.076, *p* = 0.007	Supported
H4a	IQ → PV → SCI	IE = 0.090,CI [0.068, 0.115]	Supported
H4b	PPA → PV → SCI	IE = 0.126,CI [0.089, 0.169]	Supported
H4c	PPC → PV → SCI	IE = 0.183,CI [0.128, 0.246]	Supported

### 3.2 Common method bias and multicollinearity tests

Data were collected using a questionnaire survey. To avoid common method bias, the questionnaire was completed anonymously, and Harman's single-factor test was used for examination. The results showed that a total of 10 common factors with eigenvalues greater than 1 were extracted from the unrotated factor analysis, and the variance explained by the first common factor is 23.424%, which is lower than the critical standard of 40%, indicating that there is no serious common method bias. At the same time, the VIF values of all predictor variables range from 1.118 to 1.257, all of which are less than 5, indicating that there is no serious multicollinearity problem, and the data are suitable for subsequent analysis.

### 3.3 Differences in college students’ sports consumption intention across demographic variables

This study employed independent-samples t-tests and one-way analysis of variance (ANOVA) to examine differences in sports consumption intention among college students with different demographic characteristics. The results showed that differences in sports consumption intention among college students by gender, grade, place of origin, academic discipline, interest in basketball, favorite player, monthly allowance, and monthly sports expenditure did not reach statistical significance (*p* > 0.05). Regarding RQ1, no statistically significant differences in NBA-related sports consumption intention were observed across the demographic, socioeconomic, or basketball-related characteristics examined in this study.

### 3.4 Correlation analysis of NBA stars’ China tours, perceived value, and college students’ sports consumption intention

Pearson correlation analysis was conducted to examine the relationships among information quality, players’ professional achievements, players’ personal charisma, perceived value, and sports consumption intention. The results showed that all five variables were significantly and positively correlated with each other, reaching statistical significance at the 0.01 level. Specifically, information quality was significantly and positively correlated with sports consumption intention, with a correlation coefficient of 0.349. Players’ professional achievements were significantly and positively correlated with sports consumption intention, with a correlation coefficient of 0.298. Players’ personal charisma was also significantly and positively correlated with sports consumption intention, with a correlation coefficient of 0.230. These findings indicate that the more positively college students perceive the information quality, professional achievements, and personal charisma of basketball stars, the stronger their sports consumption intention. In addition, the correlation coefficient between perceived value and sports consumption intention was 0.480, indicating a relatively strong positive association. Overall, the significant correlations among the core variables provided preliminary evidence of theoretically consistent associations and a basis for the subsequent integrated path analysis and bootstrap tests of indirect associations.

### 3.5 Hypothesis testing and mediation analysis

A single integrated path model was estimated to examine the hypothesized associations while accounting simultaneously for all three external cues. Information quality (*β* = 0.259, *p* < 0.001), players’ professional achievements (*β* = 0.202, *p* < 0.001), and players’ personal charisma (*β* = 0.200, *p* < 0.001) were each positively associated with perceived value, supporting H1a–H1c. Perceived value was positively associated with sports consumption intention (*β* = 0.356, *p* < 0.001), supporting H2. Information quality (*β* = 0.194, *p* < 0.001), players’ professional achievements (*β* = 0.124, *p* < 0.001), and players’ personal charisma (*β* = 0.076, *p* = 0.007) also remained positively associated with sports consumption intention when estimated simultaneously, supporting H3a–H3c. The three external cues jointly explained 20.5% of the variance in perceived value, while the full model explained 29.3% of the variance in sports consumption intention.

Bias-corrected bootstrap analysis further showed significant specific indirect associations through perceived value for information quality [B = 0.090, 95% BC CI (0.068, 0.115)], players’ professional achievements [B = 0.126, 95% BC CI (0.089, 0.169)], and players’ personal charisma [B = 0.183, 95% BC CI (0.128, 0.246)]. Because none of the confidence intervals included zero, H4a–H4c were supported. The corresponding direct associations with sports consumption intention also remained statistically significant in the integrated model.

## 4 Discussion

Based on the Stimulus–Organism–Response (S-O-R) theory and the Elaboration Likelihood Model (ELM), this study constructed a theoretical model in which information quality, players’ professional achievements, and players’ personal charisma were treated as antecedent variables, perceived value as the mediating variable, and college students’ sports consumption intention as the outcome variable. The hypotheses were tested using an integrated path model, with bias-corrected bootstrap confidence intervals used to evaluate the specific indirect associations. The main findings and discussion are presented as follows:

### 4.1 Differences in NBA-related sports consumption intention across demographic characteristics

Differences in sports consumption intention among college students of different genders, grades, places of origin, academic disciplines, levels of basketball interest, favorite players, monthly allowance levels, and monthly sports expenditure levels did not reach statistical significance. Regarding RQ1, no statistically significant differences in NBA-related sport consumer behavioral intention were detected across the demographic, socioeconomic, and basketball-related characteristics examined in the present study. This finding differs from some traditional studies on sports consumption. Previous research has often found that factors such as gender and income have significant effects on consumption behavior (3). However, some studies have reported findings similar to those of the present study. For example, Chen and Lin found in their study of sports event consumption that demographic variables such as gender and monthly income had weak explanatory power for game attendance intention and licensed merchandise purchase intention, and did not reach statistical significance in the validation sample, suggesting that sports consumption intention is not necessarily significantly influenced by demographic characteristics (5). The 2020 Report on Public Fitness Behavior and Consumption also indicated that differences in city, gender, age, and household income had relatively limited effects on physical sports goods consumption (27). A possible explanation is that the specific context of NBA star tours may reduce observable differences across broad demographic categories. However, the absence of statistically significant group differences does not indicate that demographic characteristics are generally irrelevant. It only suggests that no significant differences were detected under the present sample composition, measurement approach, and research context. Future studies may further examine whether demographic characteristics or basketball involvement operate as moderators of the relationships proposed in the present model.

In addition, this result may also be related to the sample composition of the present study. College students in universities in Chongqing were relatively concentrated in terms of basketball interest and favorite players, and respondents included in the final sample had prior awareness of NBA star tours in China, although they differed in their level of interest in basketball.

### 4.2 Positive relationships between core variables and sports consumption intention

The analysis results showed that information quality, players’ professional achievements, players’ personal charisma, perceived value, and sports consumption intention were all significantly and positively correlated. Specifically, the correlation coefficients between information quality, players’ professional achievements, players’ personal charisma, and sports consumption intention were 0.349, 0.298, and 0.230, respectively. This indicates that the more positively college students evaluate the information content of NBA stars’ China tours, players’ professional performance, and players’ personal image, the stronger their sports consumption intention (28, 29). The correlation coefficient between perceived value and sports consumption intention was 0.480, indicating a close relationship between value judgment and consumption intention (10, 30).

This pattern is also consistent with the theoretical expectations of the S-O-R framework and the Elaboration Likelihood Model. Higher information quality may provide college students with a clearer basis for understanding event arrangements, participation methods, and related content. Evaluations of players’ professional achievements and personal charisma may likewise be relevant to psychological responses associated with professional authority, role-model significance, affinity, and image attractiveness. However, given the cross-sectional design, the observed relationships should not be interpreted as evidence that these factors causally determine college students’ sports consumption intention.

### 4.3 The role of perceived value in sports consumption intention

The integrated path analysis showed significant indirect associations through perceived value between all three external cues and sports consumption intention. The unstandardized indirect associations were 0.090 for information quality [95% BC CI (0.068, 0.115)], 0.126 for players’ professional achievements [95% BC CI (0.089, 0.169)], and 0.183 for players’ personal charisma [95% BC CI (0.128, 0.246)]. Importantly, these associations were estimated within a single model in which the three external cues were considered simultaneously. The corresponding direct associations with sports consumption intention also remained statistically significant.

This finding suggests that college students’ sport consumption intention is associated not only with their evaluations of tour-related information and athlete attributes but also with whether they perceive the related activities, products, and services as worthwhile. Arai et al. proposed that athlete brand image includes not only athletic performance, but also dimensions such as attractive appearance and a marketable lifestyle (4), which is consistent with the inclusion of players’ professional achievements and personal charisma as two variables in this study. Kwon et al. found in their study of team-licensed merchandise consumption that perceived value played a mediating role between team identification and purchase intention (12). Shapiro et al. also found a close relationship among identification, perceived value, and purchase intention in their study of pay-per-view sports consumption (11). Taken together, these findings are consistent with the hypothesized mediating role of perceived value between tour-related external cues and sports consumption intention. However, because the external cues, perceived value, and sports consumption intention were measured concurrently, the observed indirect effects do not establish the temporal ordering or causal process implied by a mediation mechanism.

## 5 Recommendations

The findings of this study provide several evidence-based practical implications for the design and promotion of NBA stars’ China tours. Because information quality, players’ professional achievements, and players’ personal charisma were all significantly and positively associated with sports consumption intention, and significant indirect associations through perceived value were observed for all three external cues, event organizers should focus not only on increasing the visibility of tour activities but also on how communication-related and athlete-related cues are related to consumers’ value evaluations. Given the cross-sectional design of this study, the following implications should be interpreted as empirically informed priorities for practice rather than as causally established effects.

First, the information dissemination mechanism for NBA stars’ China tours should be improved. The positive associations of information quality with perceived value and behavioral intention suggest that tour-related information should be complete, timely, consistent, accessible, and relevant. Organizers may establish a centralized information platform that clearly presents event dates, locations, eligibility requirements, registration procedures, ticketing policies, activity content, livestream arrangements, and opportunities for interaction. Such measures should aim not merely to increase promotional exposure but also to reduce information uncertainty and enable college students to determine whether and how they can participate in relevant activities.

Second, communication and activity content for NBA stars’ China tours should present the participating players’ professional achievements in ways that are both accessible and verifiable. Given the significant positive associations of players’ professional achievements with perceived value and behavioral intention, organizers may highlight career milestones, competitive records, technical strengths, training practices, professional attitudes, and team experiences. Basketball demonstrations, skills clinics, training sessions, and analyses of representative performances may help translate abstract star status into more concrete and favorable value evaluations. Such content should be grounded in accurate professional information rather than exaggerated celebrity promotion.

Third, activity design should provide college students with authentic opportunities to experience the participating players’ personal charisma. In the integrated model, players’ personal charisma remained positively associated with sports consumption intention, and a significant indirect association through perceived value was also observed. These findings highlight the practical relevance of translating athlete appeal into activity experiences that consumers perceive as valuable. Organizers may incorporate moderated question-and-answer sessions, campus dialogues, basketball challenges, training demonstrations, player feedback on student-created basketball content, and carefully designed co-creation activities. These formats should emphasize meaningful interaction, enjoyment, psychological closeness, and social participation rather than simply extending the duration of athlete exposure.

Fourth, the design of tour-related activities, products, and services should be guided by the multidimensional nature of perceived value. Organizers may enhance functional value by providing practically useful basketball content, reliable event organization, clear participation procedures, and high-quality services; emotional value through enjoyable, memorable, and immersive experiences; and social value through fan-community interaction, peer participation, identity expression, and opportunities for responsible content sharing. Before introducing high-priced or limited-edition offerings, organizers should assess whether the expected benefits are commensurate with the monetary, time, and effort costs required of college students.

Finally, in the present sample, no statistically significant differences in behavioral intention were observed across gender, grade level, urban–rural background, academic major, interest in basketball, favorite player, monthly allowance, or monthly sports expenditure. Organizers should therefore avoid assuming that college students’ consumption intentions can be reliably inferred from these broad characteristics alone. Rather than developing fundamentally different strategies solely on the basis of gender, grade level, or spending capacity, organizers may provide an accessible core activity structure and allow participants to select optional experiences according to the types of value they seek. However, the absence of statistically significant differences does not imply that all student groups are identical; it only indicates that no significant differences were detected under the sampling and measurement conditions of the present study. Further consumer research is therefore warranted before implementing more refined segmentation strategies.

## 6 Research significance and limitations

### 6.1 Theoretical implications

First, the present study provides context-specific evidence for the applicability of athlete-brand concepts within the setting of NBA stars’ China tours. Previous athlete-brand research has identified athletic performance, personal image, and off-field characteristics as important bases of consumers’ evaluations of athletes and related behavioral outcomes (4, 20, 31). Building on this literature, the present study examines players’ professional achievements and personal charisma in an athlete-tour setting and considers these athlete-related cues alongside information quality as a communication-related cue. This contextual application highlights that consumer responses in athlete-tour marketing may be associated not only with attributes of the participating athletes but also with the communication environment surrounding the tour. In this sense, the study broadens the empirical context in which established athlete-brand concepts are examined, without claiming a theoretical extension of athlete-brand theory.

Second, the study provides additional context-specific evidence regarding the role of perceived value in sport consumption. Previous studies have established the relevance of perceived value in sport-event participation, spectator sport, team-licensed merchandise, and pay-per-view sport consumption (10–12). The present study applies this perspective to a multifaceted athlete-tour marketing context in which communication-related information, athlete characteristics, activities, products, and experiences coexist. By examining information quality, players’ professional achievements, and personal charisma within the same conceptual framework, the study shows that perceived value is associated with both communication-related and athlete-related cues. Moreover, the significant indirect effects through perceived value are consistent with the hypothesized mediating role of perceived value in this context; however, given the cross-sectional design, they do not establish a temporal or causal mediating mechanism. These findings add context-specific evidence to the perceived-value literature by demonstrating the applicability of this perspective in an athlete-tour setting, rather than proposing a new perceived-value mechanism.

Third, the study illustrates the usefulness of combining the S-O-R framework with an ELM-informed classification of external cues for organizing relationships in the specific context of NBA stars’ China tours. ELM provides a basis for distinguishing information quality as a communication-related message cue from players’ professional achievements and personal charisma as athlete-related source cues, whereas the S-O-R framework organizes the hypothesized relationships among these external stimuli, perceived value, and sports consumption intention. The observed positive associations between the external cues and perceived value, together with the significant indirect associations through perceived value, are consistent with this theoretical organization. Thus, the study provides context-specific support for using these established theoretical perspectives together as an analytical lens for athlete-tour marketing. However, because the study is cross-sectional and does not directly measure consumers’ information-processing routes, these findings should not be interpreted as a test, modification, or extension of S-O-R or ELM.

### 6.2 Limitations and future research

Although this study provides empirical evidence regarding the associations among NBA stars’ China tour-related cues, perceived value, and college students’ sports consumption intention, several limitations should be acknowledged. First, there is a limitation related to the geographical scope of the sample. The sample in this study was mainly drawn from universities in Chongqing. Although the sample size was relatively large (*N* = 1,024) and covered students from different grades, academic majors, and places of origin, caution is still needed when generalizing the findings to other cities or regions. Future studies could expand the sample to multiple cities in different regions, such as North China, East China, and South China, to examine the applicability of the conclusions of this study and to conduct further comparative research. Second, the cross-sectional design does not permit the temporal ordering of the three external cues, perceived value, and sports consumption intention to be established. Although the integrated path model identified statistically significant indirect effects through perceived value, these estimates should not be interpreted as evidence of causal mediation. Longitudinal studies could examine whether changes in evaluations of tour-related cues precede changes in perceived value and subsequent sports consumption intention, while experimental designs could provide stronger evidence regarding causal direction. Third, this study may be subject to recall bias. Because NBA stars’ China tours are intermittent, respondents may have differed in the timing of their exposure to related information or activities. The present study did not measure the specific mode, recency, frequency, or intensity of such exposure, which may have influenced respondents’ evaluations. Future research should consider both the timing and frequency of exposure and, where possible, collect data around specific NBA stars’ China tour events.

## 7 Conclusions

The results showed that information quality, players’ professional achievements, and players’ personal charisma were all significantly and positively correlated with college students’ sports consumption intention. The more positively college students evaluated the information related to NBA stars’ China tours, players’ athletic performance, and players’ personal image, the stronger their sports consumption intention. Bias-corrected bootstrap analysis showed significant indirect effects through perceived value for information quality, players’ professional achievements, and players’ personal charisma, providing statistical support for the hypothesized mediating role of perceived value. However, because the data were cross-sectional, these indirect effects do not establish temporal ordering or causal mediation. Higher evaluations of these three attributes were associated with higher perceived value, which was itself associated with stronger sports consumption intention. In addition, perceived value was significantly associated with sport consumption intention, whereas no statistically significant differences in behavioral intention were observed across the demographic, socioeconomic, and basketball-related characteristics examined in this study.

## Data Availability

The raw data supporting the conclusions of this article will be made available by the authors, without undue reservation.
