# Supplementary material for: NBA stars’ China tours and college students’ sports consumption intention: the mediating role of perceived value
Source: Front Sports Act Living. 2026 Aug 31;8:1920395. doi: 10.3389/fspor.2026.1920395 (PMC13572656; doi:10.3389/fspor.2026.1920395)
Supplement: Supplementary file 1 [file Datasheet1.pdf]

Supplementary Material

1. General Scale Diagnostics

Table 1. Normality Test

Variable	Mean	Skewness	Kurtosis
Information Quality	35.64	-0.185	-0.171
Players' Professional Achievements	18.70	-0.351	0.107
Players' Personal Charisma	11.44	-0.416	0.007
Perceived Value	35.78	-0.116	-0.154
Sports Consumption Intention	31.31	-0.248	0.083

Table 2. Reliability Test

Scale	Cronbach's alpha	Number of Items
Information Quality	0.856	10
Players' Professional Achievements	0.798	5
Players' Personal Charisma	0.819	3
Perceived Value	0.866	10
Sports Consumption Intention	0.869	9

2. Information Quality

Table 3. KMO and Bartlett's Test for Information Quality

Statistic	Value
KMO value	0.857
Bartlett's test: Approx. chi-square	3733.272
Bartlett's test: df	45
Bartlett's test: p-value	< 0.001

Table 4. Model Fit Indices

CMIN/DF	RMSEA	RMR	GFI	NFI	RFI	TLI
1.365	0.019	0.009	0.992	0.989	0.984	0.996

For the Information Quality measurement model, the chi-square to degrees-of-freedom ratio (CMIN/DF) was 1.365, which is below 3, indicating good model fit. The RMSEA value was 0.019, which is below 0.05, indicating a small approximation error and satisfactory model fit. The RMR value was 0.009, which is below 0.05, suggesting a low residual level. In addition, the GFI, NFI, RFI, and TLI values were 0.992, 0.989, 0.984, and 0.996, respectively, all exceeding the commonly used threshold of 0.900. These results indicate that the Information Quality measurement model demonstrated good overall fit.

Table 5. Convergent Validity Results

Item	Dimension	Loading	AVE	CR
A3	Completeness	0.773	0.567	0.797
A2	Completeness	0.725		
A1	Completeness	0.761		

Item	Dimension	Loading	AVE	CR
A5	Timeliness	0.747	0.559	0.717
A4	Timeliness	0.748		
A7	Customization	0.782	0.597	0.748
A6	Customization	0.763		
A10	Scope	0.809	0.584	0.808
A9	Scope	0.714		
A8	Scope	0.767		

The standardized factor loadings of all items under the dimensions of Information Quality were greater than 0.700, indicating that the items had good explanatory power for their corresponding latent variables. The AVE values for Completeness, Timeliness, Customization, and Scope were 0.567, 0.559, 0.597, and 0.584, respectively, all exceeding the criterion of 0.500. The corresponding CR values were 0.797, 0.717, 0.748, and 0.808, respectively, all exceeding the reference standard of 0.700.

Table 6. Discriminant Validity Results

Variable	Completeness	Timeliness	Customization	Scope
Completeness	**0.753**			
Timeliness	0.554**	**0.748**		
Customization	0.591**	0.599**	**0.773**	
Scope	0.539**	0.570**	0.601**	**0.764**

Note. Bold diagonal values are the square roots of AVE; off-diagonal values are inter-dimension correlations. ** $p < 0.01$.

3. Players' Professional Achievements

Table 7. KMO and Bartlett's Test for Players' Professional Achievements

Statistic	Value
KMO value	0.748
Bartlett's test: Approx. chi-square	1759.907
Bartlett's test: df	10
Bartlett's test: p-value	< 0.001

Table 8. Model Fit Indices

CMIN/DF	RMSEA	RMR	GFI	NFI	RFI	TLI
1.538	0.023	0.008	0.998	0.997	0.991	0.997

For the Players' Professional Achievements measurement model, the chi-square to degrees-of-freedom ratio (CMIN/DF) was 1.538, which is below 3, indicating good overall model fit. The RMSEA value was 0.023, which is below 0.05, indicating a small approximation error. The RMR value was 0.008, which is below 0.05, suggesting a low residual level. In addition, the GFI, NFI, RFI, and TLI values were 0.998, 0.997, 0.991, and 0.997, respectively, all exceeding the commonly used threshold of 0.900. These results indicate that the Players' Professional Achievements measurement model demonstrated good fit.

Table 9. Convergent Validity

Item	Dimension	Loading	AVE	CR
B3	Achievement Cognition	0.754	0.580	0.806
B2	Achievement Cognition	0.787		

Item	Dimension	Loading	AVE	CR
B1	Achievement Cognition	0.744	0.645	0.784
B5	Technical Ability and Professionalism	0.785		
B4	Technical Ability and Professionalism	0.821		

Table 10. Discriminant Validity Results

Variable	Achievement Cognition	Technical Ability and Professionalism
Achievement Cognition	**0.762**	
Technical Ability and Professionalism	0.554**	**0.803**

Note. Bold diagonal values are the square roots of AVE; off-diagonal values are inter-dimension correlations. ** $p < 0.01$.

Achievement Cognition and Technical Ability and Professionalism were significantly correlated, and the absolute value of the correlation coefficient was lower than the corresponding square root of AVE. This result indicates that the different dimensions were significantly associated in line with theoretical expectations while maintaining adequate distinctiveness. Therefore, the scale demonstrated good discriminant validity.

4. Players' Personal Charisma

Table 11. KMO and Bartlett's Test for Players' Personal Charisma

Statistic	Value
KMO value	0.717
Bartlett's test: Approx. chi-square	1076.770
Bartlett's test: df	3
Bartlett's test: p-value	< 0.001

The Players' Personal Charisma scale contained three items and had a unidimensional structure. In confirmatory factor analysis, a three-item single-factor model is typically a just-identified model with zero degrees of freedom; therefore, the overall fit indices have no substantive interpretive value. Accordingly, this study did not separately report the overall fit indices for the Players' Personal Charisma scale. Instead, its convergent validity was evaluated using standardized factor loadings, AVE, and CR. In addition, because Players' Personal Charisma was specified as a unidimensional construct, within-scale discriminant-validity testing across multiple dimensions was not applicable. Its discriminant validity relative to the other focal constructs was subsequently assessed as part of the HTMT analysis reported in Table 22.

Table 12. Convergent Validity Results

Item	Dimension	Loading	AVE	CR
C3	Personal Charisma	0.799	0.602	0.819
C2	Personal Charisma	0.741		
C1	Personal Charisma	0.786		

The standardized factor loadings of the items in the Players' Personal Charisma scale were 0.799, 0.741, and 0.786, respectively, all exceeding 0.700. This indicates that the items had good explanatory power for the latent variable of Personal Charisma. The AVE value for Players' Personal Charisma was 0.602, exceeding the criterion of 0.500, and the CR value was 0.819, exceeding the reference standard of 0.700. These results indicate that the scale demonstrated good convergent validity and composite reliability.

5. Perceived Value

Table 13. KMO and Bartlett's Test for Perceived Value

Statistic	Value
KMO value	0.895
Bartlett's test: Approx. chi-square	3827.247
Bartlett's test: df	45
Bartlett's test: p-value	< 0.001

Table 14. Model Fit Indices

CMIN/DF	RMSEA	RMR	GFI	NFI	RFI	TLI
1.238	0.015	0.008	0.992	0.990	0.986	0.997

For the Perceived Value measurement model, the chi-square to degrees-of-freedom ratio (CMIN/DF) was 1.238, which is below 3, indicating good overall model fit. The RMSEA value was 0.015, which is below 0.05, indicating a small approximation error. The RMR value was 0.008, which is below 0.05, suggesting a low residual level. In addition, the GFI, NFI, RFI, and TLI values were 0.992, 0.990, 0.986, and 0.997, respectively, all exceeding the commonly used threshold of 0.900. These results indicate that the Perceived Value measurement model demonstrated satisfactory overall fit.

Table 15. Convergent Validity

Item	Dimension	Loading	AVE	CR
F2	Functional Value	0.691	0.509	0.674
F1	Functional Value	0.735		
F7	Emotional Value	0.748	0.538	0.853
F6	Emotional Value	0.710		
F5	Emotional Value	0.753		
F4	Emotional Value	0.725		
F3	Emotional Value	0.729		
F10	Social Value	0.722	0.551	0.786
F9	Social Value	0.732		
F8	Social Value	0.771		

Table 16. Discriminant Validity

Variable	Functional Value	Emotional Value	Social Value
Functional Value	**0.713**		
Emotional Value	0.646**	**0.734**	
Social Value	0.625**	0.584**	**0.742**

Note. Bold diagonal values are the square roots of AVE; off-diagonal values are inter-dimension correlations. ** $p < 0.01$.

6. Sports Consumption Intention

Table 17. KMO and Bartlett's Test for Sports Consumption Intention

Statistic	Value
KMO value	0.860
Bartlett's test: Approx. chi-square	4268.347
Bartlett's test: df	36
Bartlett's test: p-value	< 0.001

Table 18. Model Fit Indices

CMIN/DF	RMSEA	RMR	GFI	NFI	RFI	TLI
1.523	0.023	0.012	0.992	0.991	0.987	0.996

For the Sports Consumption Intention measurement model, the chi-square to degrees-of-freedom ratio (CMIN/DF) was 1.523, which is below 3, indicating good overall model fit. The RMSEA value was 0.023, which is below 0.05, indicating a small approximation error. The RMR value was 0.012, which is below 0.05, suggesting a low residual level. In addition, the GFI, NFI, RFI, and TLI values were 0.992, 0.991, 0.987, and 0.996, respectively, all exceeding the commonly used threshold of 0.900. These results indicate that the Sports Consumption Intention measurement model demonstrated satisfactory overall fit.

Table 19. Convergent Validity

Item	Dimension	Loading	AVE	CR
Q3	Recommendation Intention	0.823	0.635	0.839
Q2	Recommendation Intention	0.783		
Q1	Recommendation Intention	0.783		
Q6	Participation Intention	0.801	0.645	0.845
Q5	Participation Intention	0.802		
Q4	Participation Intention	0.806		
Q9	Premium Purchase Intention	0.769	0.615	0.828
Q8	Premium Purchase Intention	0.791		
Q7	Premium Purchase Intention	0.793		

Table 20. Discriminant Validity

Variable	Recommendation Intention	Participation Intention	Premium Purchase Intention
Recommendation Intention	**0.797**		
Participation Intention	0.540**	**0.803**	
Premium Purchase Intention	0.567**	0.577**	**0.784**

Note. Bold diagonal values are the square roots of AVE; off-diagonal values are inter-dimension correlations. ** p < 0.01.

7. Descriptive Statistics and Construct-Level Discriminant Validity

Table 21. Descriptive Statistics of the Scales

Variable	N	Minimum	Maximum	Mean	Standard Deviation
Information Quality	1024	19	50	35.64	5.390
Players' Professional Achievements	1024	7	25	18.70	2.992
Players' Personal Charisma	1024	4	15	11.44	2.040

Variable	N	Minimum	Maximum	Mean	Standard Deviation
Perceived Value	1024	20	50	35.78	5.003
Sports Consumption Intention	1024	12	45	31.31	5.246

Table 22. HTMT Ratios Among the Focal Constructs

Construct	IQ	PPA	PPC	PV	SCI
Information Quality					
Players' Professional Achievements	0.278				
Players' Personal Charisma	0.147	0.299			
Perceived Value	0.383	0.373	0.334		
Sports Consumption Intention	0.405	0.358	0.272	0.553	

Note. IQ = Information Quality; PPA = Players' Professional Achievements; PPC = Players' Personal Charisma; PV = Perceived Value; SCI = Sports Consumption Intention.

As shown in Table 22, the HTMT values among the five focal constructs ranged from 0.147 to 0.553. The highest HTMT value was 0.553 between perceived value and sports consumption intention. All values were below the conservative threshold of 0.85, indicating adequate discriminant validity among information quality, players' professional achievements, players' personal charisma, perceived value, and sports consumption intention.

8. Final Questionnaire Items and Measurement Sources

Table 23. Final Questionnaire Items and Measurement Sources

Construct	Dimension / Item	Final Item Wording	Measurement Source(s)
Information Quality	Completeness (A1)	I can obtain the complete itinerary for NBA stars' China tours through official channels.	Refs. 1–3
Information Quality	Completeness (A2)	Information about NBA stars' China tours (e.g., player introductions and participation procedures) is comprehensive.	Refs. 1–3
Information Quality	Completeness (A3)	I can easily find information about previous NBA stars' China tour activities.	Refs. 1–3
Information Quality	Timeliness (A4)	The latest updates about NBA stars' China tours are provided in a timely manner.	Refs. 1–3
Information Quality	Timeliness (A5)	I can obtain information about livestreams or replays of NBA stars' China tours as soon as it becomes available.	Refs. 1–3
Information Quality	Customization (A6)	Relevant platforms recommend NBA stars' China tour content based on my interests.	Refs. 1–3
Information Quality	Customization (A7)	I receive personalized information about NBA stars' China tours that matches my preferences.	Refs. 1–3
Information Quality	Scope (A8)	Information about NBA stars' China tours covers multiple locations or types of activities.	Refs. 1–3
Information Quality	Scope (A9)	I can obtain information about NBA stars' China tours involving different players and different years.	Refs. 1–3
Information Quality	Scope (A10)	The available information covers not only the tour activities themselves but also public-welfare initiatives, cultural exchange, and brand interactions.	Refs. 1–3
Players' Professional Achievements	Achievement Cognition (B1)	I consider the player's NBA career to be highly successful.	Refs. 4–5
Players' Professional Achievements	Achievement Cognition (B2)	I consider the honors received by the player to be highly prestigious.	Refs. 4–5
Players' Professional Achievements	Achievement Cognition (B3)	I consider the player to hold an important place in basketball history.	Refs. 4–5
Players' Professional Achievements	Technical Ability and Professionalism (B4)	I consider the player to possess elite basketball skills.	Refs. 4–5
Players' Professional Achievements	Technical Ability and Professionalism (B5)	I highly recognize the professionalism demonstrated by the player.	Refs. 4–5
Players' Personal Charisma	Personal Charisma (C1)	I find the player's appearance and overall demeanor attractive.	Refs. 4–5
Players' Personal Charisma	Personal Charisma (C2)	I find the player approachable in public appearances.	Refs. 4–5
Players' Personal Charisma	Personal Charisma (C3)	I like the personal style that the player presents on social media.	Refs. 4–5
Perceived Value	Functional Value (F1)	NBA stars' China tours help me better understand basketball techniques and tactics.	Ref. 6
Perceived Value	Functional Value (F2)	NBA stars' China tour activities make me pay more attention to basketball culture.	Ref. 6
Perceived Value	Emotional Value (F3)	Participating in NBA stars' China tours, either online or offline, makes me feel excited.	Ref. 6

Construct	Dimension / Item	Final Item Wording	Measurement Source(s)
Perceived Value	Emotional Value (F4)	Interacting with my idol through NBA stars' China tours, either online or offline, gives me a sense of accomplishment.	Ref. 6
Perceived Value	Emotional Value (F5)	I am satisfied with NBA stars' performance during the tour activities.	Ref. 6
Perceived Value	Emotional Value (F6)	NBA stars' China tours help me relieve stress and experience enjoyment during participation.	Ref. 6
Perceived Value	Emotional Value (F7)	NBA stars' China tour activities are well organized, with rich and interesting content.	Ref. 6
Perceived Value	Social Value (F8)	NBA stars' China tours help me meet more people who also like basketball.	Ref. 6
Perceived Value	Social Value (F9)	Players' participation in public-welfare activities makes me feel a stronger sense of social responsibility.	Ref. 6
Perceived Value	Social Value (F10)	Purchasing products endorsed by the player can enhance my image within my social circle.	Ref. 6
Sports Consumption Intention	Recommendation Intention (Q1)	I would recommend NBA stars' China tour activities to my friends or classmates.	Refs. 7–9
Sports Consumption Intention	Recommendation Intention (Q2)	I would share information about NBA stars' China tours and related products.	Refs. 7–9
Sports Consumption Intention	Recommendation Intention (Q3)	I would encourage people around me to purchase products endorsed by the player.	Refs. 7–9
Sports Consumption Intention	Participation Intention (Q4)	If I had the opportunity to attend an NBA stars' China tour activity in person, I would be very willing to participate.	Refs. 7–9
Sports Consumption Intention	Participation Intention (Q5)	I would be willing to adjust my schedule in order to participate in an NBA stars' China tour activity.	Refs. 7–9
Sports Consumption Intention	Participation Intention (Q6)	When I need a related product, I would give priority to products endorsed by the player.	Refs. 7–9
Sports Consumption Intention	Premium Purchase Intention (Q7)	Even if ticket prices increased, I would still be willing to purchase a ticket to attend an NBA stars' China tour.	Refs. 7–9
Sports Consumption Intention	Premium Purchase Intention (Q8)	I would be willing to pay a higher price for limited-edition products endorsed by the player.	Refs. 7–9
Sports Consumption Intention	Premium Purchase Intention (Q9)	Even if a product endorsed by the player were priced higher than comparable products on the market, I would still be willing to purchase it.	Refs. 7–9

Note. Item wording was contextually adapted to NBA stars' China tours. The cited studies informed construct and dimension selection rather than serving as verbatim item sources. Measurement-source numbers correspond to the references listed below, and item codes correspond to those used in the validity analyses reported above.

References for Table 23

1. DeLone WH, McLean ER. The DeLone and McLean model of information systems success: a ten-year update. J Manag Inf Syst. (2003) 19(4):9–30. doi: 10.1080/07421222.2003.11045748.
2. McKinney V, Yoon K, Zahedi FM. The measurement of web-customer satisfaction: an expectation and disconfirmation approach. Inf Syst Res. (2002) 13(3):296–315. doi: 10.1287/isre.13.3.296.76.
3. Wixom BH, Todd PA. A theoretical integration of user satisfaction and technology acceptance. Inf Syst Res. (2005) 16(1):85–102. doi: 10.1287/isre.1050.0042.
4. Pifer ND, Mak JY, Bae WY, Zhang JJ. Examining the relationship between star player characteristics and brand equity in professional sport teams. Marketing Management Journal. (2015) 25(2):88–106. doi: 10.63963/001c.151098.
5. Arai A, Ko YJ, Ross S. Branding athletes: exploration and conceptualization of athlete brand image. Sport Manag Rev. (2014) 17(2):97–106. doi: 10.1016/j.smr.2013.04.003.
6. Sweeney JC, Soutar GN. Consumer perceived value: the development of a multiple item scale. J Retail. (2001) 77(2):203–220. doi: 10.1016/S0022-4359(01)00041-0.
7. Ajzen I. The theory of planned behavior. Organ Behav Hum Decis Process. (1991) 50(2):179–211. doi: 10.1016/0749-5978(91)90020-T.

8. Zeithaml VA, Berry LL, Parasuraman A. The behavioral consequences of service quality. J Mark. (1996) 60(2):31–46. doi: 10.1177/002224299606000203.
9. Dong DH, Jin YF. A study on the antecedents of consumer behavioral intention. Nankai Business Review. (2003) 6(6):46–51. [in Chinese].
